# PRMT5 Promotes Cyclin E1 and Cell Cycle Progression in CD4 Th1 Cells and Correlates With EAE Severity

**DOI:** 10.3389/fimmu.2021.695947

**Published:** 2021-06-08

**Authors:** Stephanie A. Amici, Wissam Osman, Mireia Guerau-de-Arellano

**Affiliations:** ^1^ School of Health and Rehabilitation Sciences, Division of Medical Laboratory Science, College of Medicine, Wexner Medical Center, The Ohio State University, Columbus, OH, United States; ^2^ Discovery PREP Program, College of Medicine, Wexner Medical Center, The Ohio State University, Columbus, OH, United States; ^3^ Institute for Behavioral Medicine Research, The Ohio State University, Columbus, OH, United States; ^4^ Department of Microbial Infection and Immunity, The Ohio State University, Columbus, OH, United States; ^5^ Department of Neuroscience, The Ohio State University, Columbus, OH, United States

**Keywords:** PRMT5, cell cycle, relapsing-remitting experimental autoimmune encephalomyelitis, T cell, multiple sclerosis

## Abstract

Multiple Sclerosis (MS) is a debilitating central nervous system disorder associated with inflammatory T cells. Activation and expansion of inflammatory T cells is thought to be behind MS relapses and influence disease severity. Protein arginine N-methyltransferase 5 (PRMT5) is a T cell activation-induced enzyme that symmetrically dimethylates proteins and promotes T cell proliferation. However, the mechanism behind PRMT5-mediated control of T cell proliferation and whether PRMT5 contributes to diseases severity is unclear. Here, we evaluated the role of PRMT5 on cyclin/cdk pairs and cell cycle progression, as well as PRMT5’s link to disease severity in an animal model of relapsing-remitting MS. Treatment of T helper 1 (mTh1) cells with the selective PRMT5 inhibitor, HLCL65, arrested activation-induced T cell proliferation at the G1 stage of the cell cycle, suggesting PRMT5 promotes cell cycle progression in CD4^+^ T cells. The Cyclin E1/Cdk2 pair promoting G1/S progression was also decreased after PRMT5 inhibition, as was the phosphorylation of retinoblastoma. In the SJL mouse relapsing-remitting model of MS, the highest PRMT5 expression in central nervous system-infiltrating cells corresponded to peak and relapse timepoints. PRMT5 expression also positively correlated with increasing CD4 Th cell composition, disease severity and Cyclin E1 expression. These data indicate that PRMT5 promotes G1/S cell cycle progression and suggest that this effect influences disease severity and/or progression in the animal model of MS. Modulating PRMT5 levels may be useful for controlling T cell expansion in T cell-mediated diseases including MS.

## Introduction

Multiple Sclerosis (MS) is a demyelinating disease of the central nervous system (CNS) that often starts in young adulthood and currently has no cure. The majority of patients are diagnosed with relapsing-remitting MS (RRMS), characterized by episodes of disease activity and recovery. Heterogeneous symptoms include but are not limited to fatigue, vision problems, muscle weakness/paralysis, emotional changes/depression and speech/swallowing problems (1). It is understood that myelin-specific inflammatory Th1 and Th17 cells infiltrate the CNS, causing focal demyelinating plaques throughout the CNS (2, 3). Activated memory T cells play a critical role in MS active disease (4, 5), are reduced during positive response to therapy (6) and increase during periods of active disease (2, 5, 7–9). Inflammatory T cells are also tied to the onset of disease in the preclinical rodent model of MS, experimental autoimmune encephalomyelitis (EAE) (10). T cell activation is expected to result in clonal proliferation and, therefore, expansion of myelin-specific T cells which can infiltrate the CNS and promote disease activity.

Protein arginine N-methyltransferases (PRMTs) are enzymes that catalyze the addition of methyl groups onto arginines on histones and other proteins. They play a role in many cell functions including cell signaling, proliferation and differentiation (11). One way T cell responses can be dampened in EAE is through pan-methylation inhibitors. Treatment with these inhibitors has been linked to reduced EAE clinical scores and T cell responses (12–14). PRMT5 is the main protein involved in symmetric dimethylation (15). Our lab previously demonstrated that PRMT5 inhibition reduced T cell proliferation and EAE symptoms in the EAE mouse model (16, 17). However, the mechanism behind PRMT5-mediated control of T cell proliferation and whether PRMT5 changes during disease are linked to EAE disease severity or activity remains unknown.

Here, we examine the role of PRMT5 in CD4^+^ T lymphocyte cell cycle progression and the cyclin/cdk pairs that control the cell cycle. In addition, we also study how PRMT5 is linked to disease severity in an animal model of relapsing-remitting MS. We find that PRMT5 promotes the CyclinE1/Cdk2 pair and G1/S progression and that PRMT5 and Cyclin E1 expression in CNS infiltrates is significantly linked to clinical EAE disease severity. Overall, these results show that PRMT5 promotes G1/S cell cycle progression and provide a link between PRMT5 and disease severity and/or progression in the animal model of MS.

## Materials and Methods

### Mice

B10.PL (Jackson Laboratory, Bar Harbor, ME) and myelin basic protein (MBP) Ac_1-11_ T cell receptor (TCR)-transgenic mice (described in (18)) were bred and maintained in pathogen-free conditions at The Ohio State University Laboratory Animal Resources. Murine pathogen free SJL/J mice were purchased from Taconic Biosciences (Rensselaer, NY). All animal studies were performed in accordance to the guidelines of the Animal Welfare Act (AWA) and the Policy on Humane Care and Use of Laboratory Animals (PHS). All procedures for animals were approved under electronic Institutional Animal Care and Use Committee (IACUC) protocol number 2013A00000151-R1 or -R2.

### EAE

9-week SJL/J mice were subcutaneously injected at two sites on their backs with an emulsion of 0.1 mg of proteolipid peptide (PLP_139-151_, CS Bio) in complete Freund’s adjuvant supplemented with heat-killed *Mycobacterium tuberculosis* for a final concentration of 4 mg/ml (both from Difco/Sigma)/PBS per site. From day 7 through the end of the experiment, mice were monitored daily for weight and EAE score. Mice were scored by Hooke Labs scoring system; briefly, 0 - no disease, 1 – limp tail, 2 – hind limb impairment, 3 – hind limb paralysis, 4 – complete hindlimb paralysis and partial front limb paralysis, 5 – death. This is a relapsing-remitting form of EAE. Mice were deemed in remission when their score dropped by at least 1.5 from peak. Mice were considered in relapse when their score rose by at least 1 from lowest score in remission. Mice were sacrificed at various stages of disease, including peak, remission and relapse. Sham mice anesthetized and injected with PBS were used as controls. Mice were euthanized by injection with 25 mg/ml ketamine and 4 mg/ml xylazine (150 ml/20 g mouse) and perfused with PBS. Brains and spinal cords were combined together (termed CNS throughout the paper) from representative mice and processed as described in (16) for flow cytometry and immunoblotting studies.

### MBP TCR Transgenic T Helper 1 Cells

Murine T helper 1 (Th1) cells were generated from MBP TCR-transgenic mice (18) as described in (16). Cells were subsequently maintained by MBP_Ac1-11_ stimulation (CS Bio) and irradiated splenocytes in the presence of rhIL-2 (Miltenyi) every 7-10 days. Flow cytometry and ELISA characterization of Th1 cells cytokine profile showed that they were 98% positive for the Th1 cytokine IFN-γ (**Figure S1**). No IL-4 was detectable in Th1 activated cells supernatant (not shown). Cells collected at 7-10 days served as resting cells. For experiments, T cells were activated on anti-CD3/-CD28 coated wells (1µg/ml of each antibody, eBioscience, 16-0031-86 and 16-0281-86) to avoid having non-T cells in the experiments. Cells were plated in the presence of vehicle control or HLCL65 (developed at OSU) or, for proliferation and Il-2 experiments, with Epizyme EPZ015666 (Selleck) (at 5 uM for various lengths of time as described in the figure legends).

### Immunoblotting

Resting or activated T cells were collected at various timepoints and the cell pellets were frozen at -80°C until analysis. At that time, cells were lysed in RIPA buffer (10 mM Tris pH 7.4, 150 mM NaCl, 1% Triton X-100, 0.1% SDS, 1% deoxycholate) containing protease and phosphatase inhibitors (Thermo Fisher Scientific). A BCA assay (Thermo Fisher Scientific) was run to determine protein concentrations and 5-20 µg of protein was run per sample on a 10% or 14% SDS-PAGE gel and transferred to PVDF or nitrocellulose membrane. Blots were incubated with Odyssey blocking buffer (Li-cor) or 1% non-fat milk in TBS with 0.05% Tween-20 for 30 min and then probed with primary antibodies for 3 h at room temperature or overnight at 4°C in blocking buffer or 1% non-fat milk in TBS with 0.05% Tween-20. Primary antibodies: PRMT5 (Abcam ab31751, 1:1000), H4R3me2s (MilliporeSigma SAB4300870, 1:500), pRb (Cell Signaling 2181s, 1:250), Rb (Santa Cruz SC102, 1:100), Cyclin E1 (Cell Signaling 20808s, 1:500), Cdk2 (Cell Signaling 2546s, 1:200), Cdk4 (Santa Cruz sc-56277, 1:100), Cyclin D1 (Abcam ab134175, 1:200) and β-actin (Sigma-Aldrich A1978, 1:50,000). Blots were then washed and incubated in secondary antibodies (donkey anti-rabbit 800CW and donkey-anti-mouse 680RD, Licor) at 1:20,000 each, 1h, RT. An Odyssey-CLx (Li-Cor) was used to image all blots and Image Studio software (Li-Cor) was used to quantify results.

CNS infiltrating cell protein from individual mice or pooled from multiple mice (for conditions with low infiltrating CNS cell numbers) were used as samples for protein analyses. A minimum of ~400,000 cells per sample were solubilized for protein analyses. 5-15 ug of protein were loaded for analyses. Equal amounts of protein were loaded and analyzed in individual experiments and normalized to sham. Two to three experiments were combined (**Figures 3C–I**).

### Flow Cytometry

Isolated CNS (brain and spinal cord) infiltrating cells were used directly *ex vivo* for flow cytometric analyses. Cells were incubated in Fc block (Biolegend 101320, TruStain FcX) for 10 min at 4°C and then primary antibodies for 15 min at 4°C. Primary antibodies included: CD3 (BioLegend, 100334, clone 145-2C11), CD4 (BioLegend, 100531 or eBioscience, 12-0042-85, clone RM4-5). Samples were run on a FACSCalibur with DxP multicolor upgrades (Cytek Biosciences, Fremont, CA) and analyzed using FlowJo software (FlowJo, Ashland, OR).

### Proliferation Assay

mTh1 cells were plated at 100,000-125,000 cells/well in triplicate on anti-CD3/-CD28 coated plates with either DMSO vehicle control, 5µM HLCL65 or 5µM EPZ015666. Cells were pulsed with 1µCi of tritiated (^3^H)-thymidine at 6, 30 or 56h post-plating and harvested at 24, 48 or 72h onto a Unifilter-96 well plate (PerkinElmer, catalog no. 6005174). MicroScint-20 scintillation fluid was added and plates were run on a TopCount (PerkinElmer).

### BrdU

mTh1 cells (0.5-1x10^6^) were plated on anti-CD3/CD28 coated 48-well plates for 48h in the presence of DMSO or 2.5µM HLCL65. Cells were incubated with 10µM BrdU for the last 4 h at 37°C (BrdU APC flow kit, BD 552598). For controls, 3µM aphidocolin for G1/S arrest or 0.25µM paclitaxel for G2/M arrest were added 15 min prior to addition of BrdU. Cells were stained as described in manufacturer’s instructions. Briefly, cells were blocked 10 min at 4°C in FC block (Biolegend). Primary antibodies, CD4-e450 (48-0042-82, eBioscience clone RM4-5) and CD44-FITC (103006, Biolegend, clone IM7) were added and incubated 15 min at 4°C. Cells were washed, fixed with BD Fixation Solution for 20 min at 4°C, then washed with BD Perm Wash and stored overnight in FACS. The next day, they were washed in BD Perm Wash, incubated in BD CytoPerm Permeabilization Buffer Plus 10 min at 4°C, refixed with BD Fixation solution, 5 min at 4°C, incubated with DNase solution 1h at 37°C, washed and stained with BrdU 20 min at RT, washed and resuspended with 7-AAD to stain total DNA. Cells were then run on a FACSCanto flow cytometer (BD) and analyzed by FlowJo software.

### ELISA

Supernatants from mTh1 cells plated at 5x10^6^ cells/24-well were collected at various timepoints poststimulation and analyzed by sandwich ELISA. Mouse IL-2 reagents were from eBioscience [recombinant (14-8021-64), biotinylated (13-7021-85), purified (14-7022-85)]. ELISA was performed as previously described (19).

### Statistics

All statistical analyses were performed using GraphPad Prism software. Specific analyses are described in the figure legend of each figure. Generally, student’s t test or 1-way ANOVA followed by Sidak’s post-hoc multiple comparisons test was used as appropriate. For correlation analyses, Pearson correlation was used for parametric data (expression vs. flow percentages, expression vs. expression) and Spearman correlation was used for non-parametric data (scores vs. expression).

## Results

### PRMT5 Inhibition Arrests TCR-Induced Cell Cycle Progression at theG1/S Checkpoint

**Figure 1:**
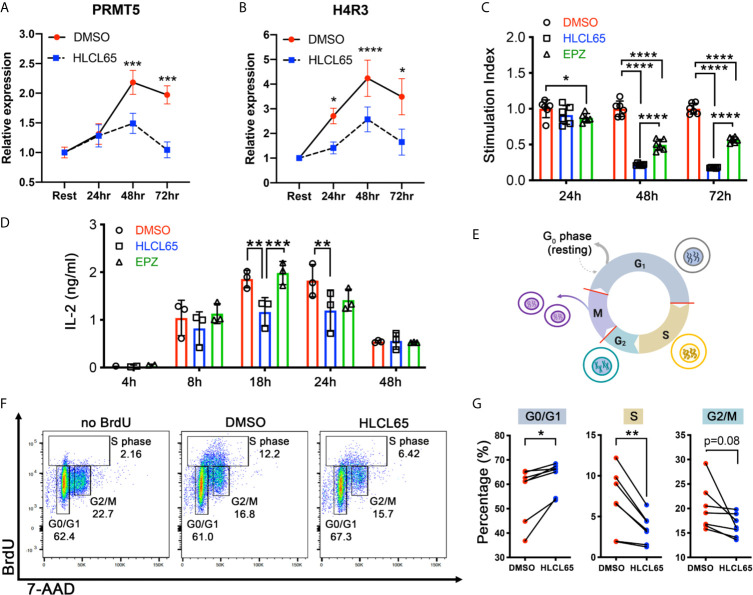
PRMT5 inhibition arrests TCR-induced cell cycle progression at the G1/S checkpoint. **(A, B)** mTh1 cells were activated on anti-CD3/CD28 coated plates, treated with 5 µM HLCL65 or vehicle control (DMSO) and harvested at the time of plating (resting) or 24, 48 or 72h post-plating. They were analyzed by immunoblot with PRMT5 **(A)** or H4R3 **(B)**. Data plotted represent relative protein expression at indicated timepoints. Plots are combined 4-6 experiments, n=8-13/timepoint/condition. **(C)** mTh1 cells were plated on anti-CD3/CD28 coated plates, treated with vehicle (DMSO), 5 μMHLCL65 or 5 μM EPZ, and pulsed with tritiated ^3^H-thymidine at 24, 48 or 72h post-plating with vehicle or HLCL65. Cells were harvested 16-18h later. This is a representative experiment with 6 technical replicates/group, analyzed by two-way ANOVA with Tukey’s multiple comparisons test. **(D)** Supernatants from mTh1 cells plated on anti-CD3/CD28 coated plates were collected at 4h, 8h, 18h, 24h and 48h ± HLCL65, EPZ or vehicle and analyzed for IL-2 cytokine expression by ELISA. Data pooled from 3 independent experiments. Data analyzed by two-way ANOVA with Tukey’s multiple comparisons test. **(E)** Diagram of the cell cycle stages including Gap 1 (G1), Synthesis (S), Gap 2 (G2) and Mitosis (M). Cells represent the stage the cell is in at each phase of the cell cycle. Red lines on the cell cycle represent cell cycle checkpoints. Created with BioRender.com, modified from a template. **(F)** Flow cytometry plots of activated mTh1 cells demonstrating the gating strategy and representative results in the negative control (no BrdU), vehicle (DMSO) or PRMT5 inhibitor (2.5 µM HLCL65). **(G)** Quantification of the results from analyses in **(F)**, corresponding to four independent experiments. Graphs for Gap 0/Gap 1 (G0/G1), Synthesis (S) and Gap 2/mitosis (G2/M). Results analyzed by paired t-test, n=7 pairs. Graphs are line or bar graphs representing mean ± SD with individual data points plotted **(A–D)** or before-after (plot individual points) **(G)**. *p < 0.05, **p < 0.01, ***p < 0.001, ****p < 0.0001.

PRMT5 promotes proliferation in mouse and human T helper (Th) cells (16, 17, 20), but the underlying mechanism is not clearly understood. To investigate this, we used myelin-specific MBP TCR transgenic mouse Th1 cells. First, we tested the impact of PRMT5 inhibition on PRMT5 expression and activity, as well as T cell proliferation and IL-2 production, after TCR stimulation. (**Figures 1A–D**). PRMT5 and its symmetric dimethylation mark (SDM) histone 4 at arginine 3 (H4R3) were upregulated after T cell activation, with a maximum at 48 hours (**Figures 1A, B**, DMSO, red). Treatment with HLCL65, a selective PRMT5 inhibitor that binds to the substrate and SAM binding pockets in PRMT5, blunted the PRMT5 and SDM expression increase observed after T cell activation (**Figures 1A, B**, HLCL65 in blue). As expected, and consistent with the decrease in PRMT5 activity, Th1 cell proliferation was robustly reduced by the selective PRMT5 inhibitor HLCL65 at both 48h and 72h (**Figure 1C**). As previously observed, HLCL65 also decreased the cytokine IL-2, a cytokine linked to T cell proliferation (**Figure 1D**). We also tested EPZ015666, a selective PRMT5 inhibitor that acts *via* a different binding mechanism, namely substrate binding competition. While EPZ015666 also reduced proliferation at all timepoints examined, it did not modulate IL-2 expression and the anti-proliferative effect was more muted compared to HLCL65 (**Figures 1C, D**), suggesting that selective PRMT5 inhibitors with distinct binding mechanisms may differentially modulate T cell function. Since the objective of this work was to understand the role of PRMT5 on T cell proliferation, we focused subsequent experiments on the HLCL65 inhibitor.

When a T cell receives a proliferative signal such as TCR stimulation *via* CD3/CD28, it progresses through several cell cycle phases, including Gap1 (G1), Synthesis (S), Gap2 (G2) and Mitosis (M) (**Figure 1E**). PRMT5 may promote the cell cycle at several of the cell cycle checkpoints (**Figure 1E**, red lines). To determine which checkpoint(s) are modulated by PRMT5 inhibition, we treated MBP-specific Th1 cells with HLCL65 for 48 hours and evaluated cell cycle progression *via* BrdU staining. Since some T cell expansion is necessary to study PRMT5’s effects on the cell cycle, we chose an intermediate concentration of HLCL65 at which 50-60% of T cell proliferation remains. HLCL65 treatment significantly increased cells in the G0/G1 phase of the cell cycle while reciprocally reducing the percent of cells in S phase and a trend to decrease in the G2/M phase (**Figures 1F, G**). These data indicate that PRMT5 activity is required for the G1 to S transition in Th cells after TCR stimulation and suggest PRMT5 is a key driver of CD4^+^ T cell progression through the cell cycle.

### PRMT5 Inhibition Suppresses Cyclin E1/CDK2 Expression and Rb Hyperphosphorylation in Inflammatory T Cells

**Figure 2:**
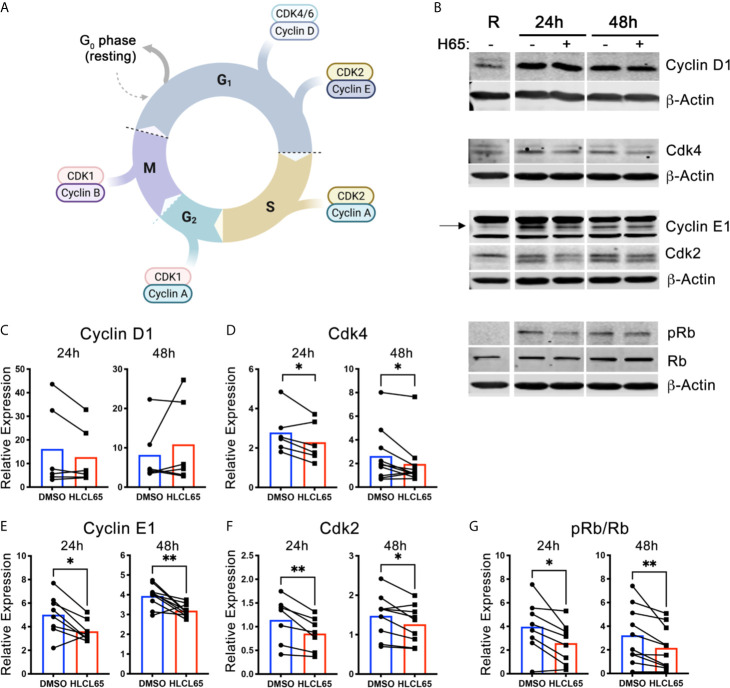
PRMT5 inhibition suppresses Cyclin E1/CDK2 expression and Rb hyperphosphorylation in inflammatory T cells. **(A)** Diagram of the expression of cyclins and CDKs at the different cell cycle stages. Created with BioRender.com, modified from a template. **(B–G)** mTh1 cells were activated on anti-CD3/CD28 coated plates, treated with 5 µM HLCL65 or vehicle control (DMSO) and harvested at various timepoints for protein analyses. **(B)** Immunoblots of mTh1 cells probed with cell cycle proteins at resting, 24h and 48h post-plating. **(C–G)** Protein quantification of **(C)** Cyclin D1, **(D)** Cdk4, **(E)** Cyclin E1, **(F)** Cdk2, **(G)** ratio of phosphorylated Rb at S608 to total Rb. DMSO values are relative to resting. Data pooled from 4-6 independent experiments, n=6-10, student’s paired t-test. *p < 0.05, **p < 0.01.

Cell cycle progression through the G1/S checkpoint is controlled by the Cyclin D1/Cdk4/6 and Cyclin E1/Cdk2 pairs [(21), **Figure 2A**]. Cyclin expression is induced by mitogenic signals, and the resulting cyclins associate with and activate their Cdk partner. Early in G1 phase, the Cyclin D1/Cdk4/6 complex monophosphorylates the retinoblastoma tumor suppressor protein (Rb). However, such phosphorylation does not inactivate Rb. Instead, for a cell to progress to S phase, Cyclin E1/Cdk2 expression/activation must hyperphosphorylate Rb. Hyperphosphorylation inactivates Rb and results in E2F release and translocation to the nucleus, where it can induce the genes necessary for S phase. We hypothesized that PRMT5 inhibition impacts one or both of these cyclin/cdk promoters of G1/S progression and, to address that, evaluated Cyclin D1/Cdk4 and Cyclin E1/Cdk2 after HLCL65 treatment.

We first examined the early G1 proteins, Cyclin D1 and Cdk4. We found that Cyclin D1 protein expression increased from resting upon T cell activation at all time points examined (**Figures 2B, C**). However, treatment with HLCL65 did not significantly inhibit this response. On the other hand, Cdk4 expression decreased in T cells treated with HLCL65 compared to DMSO control at 24h and 48h (**Figures 2B, D**), suggesting PRMT5 may play a role in the progression through the early stages of G1 phase *via* Cdk4.

Even if Cdk4 is controlled by PRMT5, additional involvement of the late G1 CyclinE1/Cdk2 complex would be necessary to fully push the cell through the G1/S checkpoint. Therefore, we next quantified the levels Cyclin E1 and Cdk2 in PRMT5-inhibited mTh1 cells and controls. Cyclin E1 was increased after activation in the absence of HLCL65, and was significantly decreased after HLCL65 treatment (**Figures 2B, E**). Similarly, Cdk2 was also decreased after HLCL65 treatment (**Figures 2B, F**). The binding of Cyclin E1 to Cdk2 activates Cdk2, which hyperphosphorylates Rb and allows cell cycle progression into S phase. Our analysis showed an increase in S608 Rb phosphorylation, a phosphorylation mark linked to hyperphosphorylation, when T cells were activated. Conversely, PRMT5 inhibitor treatment reduced the ph-Rb^S608^/total Rb phosphorylation ratio (**Figures 2B, G**). Collectively, our results indicate that PRMT5 drives Cyclin E1/Cdk2 and Rb hyper-phosphorylation, two crucial elements that drive G1/S progression and proliferation.

### PRMT5 SDM and Cyclin E1 Expression in CNS Infiltrates Correlate With RR EAE Disease Progression

**Figure 3:**
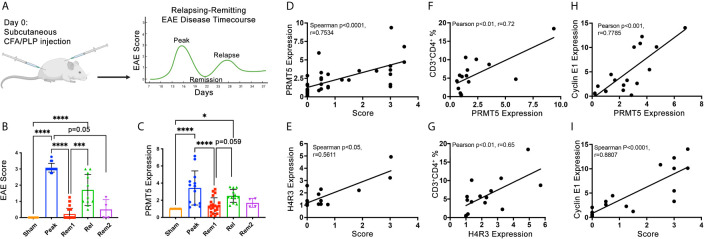
PRMT5, SDM and Cyclin E1 expression in CNS infiltrates correlate with RR EAE disease severity and/or progression. **(A)** Experimental design diagram: graphical representation of the typical EAE course in SJL mice injected with PLP_(139-151)_. Created with BioRender.com. For actual EAE data in mice used for data in B through I, please see **Figure S3**. **(B)** Mean scores calculated from mice injected with PBS instead of PLP (sham), mice collected at peak disease [day **(D)**14], in remission 1 (considered in remission if score dropped ≥1.5, D19-D25), in relapse (considered in relapse if score dropped by 1.5 or more and then rose by 1 or more, D28-33), in remission 2 (considered rem2 if mouse went through relapse and back to remission). **(C)** CNS infiltrating cells were collected at the times described in **(B)** and analyzed by immunoblot for PRMT5. **(D–I)** CNS infiltrating cell protein from individual mice or pooled from multiple mice (for conditions with low infiltrating CNS cell numbers) were used as samples for protein analyses of PRMT5, H4R3 and Cyclin E1 immunoblot and one aliquot was analyzed by flow cytometry for immune cell composition (and correlation analyses were performed. CNS infiltrating cell protein from individual mice or pooled from multiple mice (for conditions with low infiltrating CNS cell numbers) were used for protein analyses. A minimum of ~400,000 cells per sample were used and equal amounts of protein (5-15 μg) loaded and data were normalized to sham **(C–I)**. **(D, E)** Spearman correlation of score vs. **(D)** PRMT5 or **(E)** H4R3 expression. **(F, G)** Pearson correlations were done between CD3^+^CD4^+^ T cell % and **(F)** PRMT5 or **(G)** H4R3 SDM expression in infiltrating CNS cells. **(H, I)** Correlation between Cyclin E1 expression and **(H)** PRMT5 expression or **(I)** score in infiltrating CNS cells. Pearson correlations were done for CD3^+^CD4^+^ T cells vs. protein expression or protein vs. protein. Spearman correlations were done for scores vs. protein expression. For **(D–I)**, n=15-36 with technical replicates from n=59-83 total mice from 2-4 independent experiments. Graphs **(B, C)** quantify relative expression normalized to actin loading control and combined from 4 independent experiments, (9-18 sham, 13-16 peak, 21-35 rem1, 11-12 relapse, 4 rem2), biological samples were kept separate if possible, but pooled for each timepoint as necessary for each experiment, 1-2 technical replicates/experiment, graphs represent mean ± SD, *p < 0.05, ***p < 0.001, ****p < 0.0001.

Active clinical signs may be linked to active proliferation of T cells in target CNS tissues. To determine whether PRMT5 and PRMT5-dependent CyclinE1 activity is linked to active clinical signs, we used the SJL experimental autoimmune encephalomyelitis (EAE) model of MS. In this model, SJL mice immunized with PLP_139-151_ peptide develop clinical signs during peak disease around day 14 and subsequently in relapses that occur after remission periods of low clinical signs (**Figure 3A** and **Figure S3**). To determine whether PRMT5 expression is linked to EAE clinical signs, we isolated CNS-infiltrating cells from the CNS in sham or PLP-immunized mice at peak disease (day 14), remission 1 (≥1.5 score drop, days D19-D25), relapse (score hike of ≥1 after remission, D28-33) and remission 2 (≥1.5 score drop after relapse or stable remission after initial peak). As expected, EAE scores were highest during peak disease and relapse phases, while sham mice and mice during remission had negative or low scores (**Figure 3B**). PRMT5 expression evaluation at these stages of disease revealed that PRMT5 expression in CNS infiltrating cells mirrors EAE disease progression (**Figure 3C**), with a significant increase from sham animals (injected with PBS) to mice at peak with severe EAE symptoms. Instead, PRMT5 expression was significantly decreased in mice collected during remission (**Figure 3C**), and a trend toward increase was observed from remission to relapse. Taken together, these data suggest PRMT5 expression in the CNS correlates with disease activity, particularly at early stages of EAE.

PRMT5 expression and activity were significantly linked to increases in disease scores taken throughout the entire course of the disease (**Figures 3D, E**). In addition, PRMT5 expression and activity in CNS infiltrating cells significantly and positively correlated with the percentage of CD4^+^ Th cells within those infiltrating cells (**Figures 3F, G**), suggesting an important contribution of CD4^+^ Th cells to PRMT5 increases in the CNS during EAE. In accordance with PRMT5 promotion of Cyclin E1 and cell cycle progression in CD4^+^ Th cells, Cyclin E1 expression also tightly and positively correlated with both PRMT5 increases in CNS infiltrating cells and disease severity (**Figures 3H, I**), suggesting that higher G1/S T cell cycle activity increases EAE severity.

## Discussion

In this manuscript, we show that pharmacological interference with PRMT5 activity suppresses expression of G1/S checkpoint CyclinE1/Cdk2 and arrests CD4 Th cells in G1/S. In addition, we find that higher PRMT5, SDM activity and Cyclin E1 expression in immune cells infiltrating the CNS is positively linked to clinical EAE disease severity. These data indicate that PRMT5 activity is necessary for proper G1/S cell cycle progression and suggests that PRMT5’s effects on cell cycle progression are a contributor to disease.

PRMT5 is overexpressed in multiple cancers, including ovarian, prostate, lung and glioblastoma, where it promotes cancer cell hyperproliferation (22–26). More recently, a role for PRMT5 in the physiological limited proliferative response that T cells undergo upon TCR stimulation has been recognized. We have shown that either selective PRMT5 inhibitors, namely CMP5 and HLCL65, or PRMT5 deletion suppress TCR-induced proliferation in CD4 Th cells (16, 17). The finding that PRMT5 controls T cell proliferation has been independently reproduced by other laboratories using animal models of PRMT5 deletion (27, 28) and/or other types of selective PRMT5 inhibitors, such as C220 (29). Here, we additionally tested the effects of EPZ015666 on Th cell proliferation and IL-2 production. Surprisingly, even though both are potent and selective PRMT5 inhibitors, we observed a more robust effect of HLCL65 on T cell activity, namely half of HLCL65’s effect on T cell proliferation and no effect on IL-2 production. This is potentially interesting from the clinical application and drug design perspective. Drugs that potently inhibit PRMT5 but have a lesser effect on immune T cell responses would be beneficial for cancer treatment while drugs that influence T cell responses may be beneficial in autoimmune and other T cell mediated diseases. The possibility that different mechanisms of PRMT5 binding/inhibition may influence T cell vs. cancer effects is intriguing. While HLCL65 binds to both the methyl-donor SAM binding pocket and the peptide substrate pockets in PRMT5, EPZ015666 only blocks substrate-binding. While exploring this question was not the focus of this work, understanding how inhibitor mechanism of PRMT5 blockade differentially modulate cancer and immune effects is worthy of further investigation.

In general, Th cell proliferation is dependent on progression through the cell cycle through the G1/S and G2/M checkpoints. However, the exact cell cycle checkpoint and mechanism impacted by PRMT5 inhibition in CD4 Th cell remained unknown. Our finding that PRMT5 activity loss impairs progression through G1/S is consistent with other work in breast, hepatic and medulloblastoma cancer cells (30–34). There is also one report that the selective PRMT5 inhibitor, C220, impairs G1/S cell cycle progression in CD3 lymphocytes containing CD4 Th cells (29). PRMT5 deletion in the entire hematopoietic compartment was also associated with a G1/S arrest (35, 36). While studies about the mechanisms by which PRMT5 regulates the cell cycle in cancer can be helpful to infer mechanisms in untransformed Th cells, cancer cells harbor multiple mutations impacting cell cycle checkpoints. Therefore, the validity of hypotheses based on transformed cell data need to be validated with primary CD4 Th cells. Indeed, while Cyclin D1 has been shown to be suppressed by PRMT5 inhibition in cancer cells (37), Cyclin D1 was unaffected by PRMT5 inhibitor treatment in untransformed Th cells, even though it is induced upon TCR stimulation (**Figures 2A–C**). Rather, PRMT5 inhibitor treatment in Th cells suppressed expression of Cyclin E1 and its partner Cdk2 and resulted in hyperphosphorylation of Rb. Our results of a positive effect of PRMT5 on Cyclin E1 is in contrast with studies that have found PRMT5 forms part of the Cyclin E1 repressive complex (CERC) that restrains Cyclin E1 expression. These differences are worth investigating in future studies, although they may be simply be due to differences in cell type and activation stimuli.

A critical modulator and activator of Th cell proliferation is the T cell growth cytokine IL-2. IL-2 is produced by activated T cells and promotes growth and proliferation in an autocrine manner. Interestingly, early studies showed that PRMT5 knockdown repressed IL-2 production (Richard ref) in Jurkat T cells and we have subsequently shown that PRMT5 inhibition or deletion suppresses IL-2 production in primary T cells. Therefore, PRMT5 may control T cell proliferation through control of IL-2 production, although direct effects on the cell cycle could additionally play a role. The T cell model used in this study were Th1 cells, which produce large amounts of IL-2 upon T cell stimulation. In contrast, IL-2 is generally undetectable in the supernatants of activated Th2 cells, which are still sensitive more resistant to proliferation inhibition with PRMT5 inhibitors. This suggests that control of IL-2 is one of the major mechanisms by which PRMT5 controls T cell proliferation. However, high dose PRMT5 inhibitors may affect proliferation independently of IL-2 as seen in Th2 cells. It would be interesting to evaluate whether similar PRMT5 inhibition cell cycle and cyclin changes as those observed in Th1 cells occur in Th2 cells, which produce much lower amounts of IL-2. Interestingly, effects of PRMT5 inhibition on IL-2 secretion are already observable at 8 hours, prior to substantial induction of PRMT5 in T cells. This suggests that the low basal levels of PRMT5 expression may result in the PRMT5 activity that results in IL-2 production. This raises the question of how T cells suppress these effects prior to T cell activation. We speculate this may occur *via* changes in PRMT5 localization or differential complex partners. Future studies would be needed to directly address these hypotheses.

PRMT5 methylates a number of histone, splicing, transcription factor and other protein targets, any of which could directly or indirectly contribute to Cyclin E1/Cdk2 expression and proliferation. In cancer cells, a number of cell cycle mediators have been shown to interact with, be directly methylated by or be regulated by PRMT5. For instance, PRMT5 overexpression has been shown to increase CDK4 levels (30) similar to the increased Cdk4 expression observed in murine T cells at timepoints at which PRMT5 expression is induced. HLCL65 treatment suppressed this induction, suggesting Cdk4 induction in Th cells is dependent of PRMT5 activity. In addition, human CDK4 has been shown to interact with PRMT5, promoting Rb phosphorylation and regulation of E2F-dependent gene transcription (30). E2F itself has been reported to be methylated by PRMT5 on arginines R111 and R113, which promotes cell proliferation (38, 39). Therefore, PRMT5’s impacts on cell proliferation may be the result of changes in multiple PRMT5 targets. There is much less information of PRMT5 and Cyclin E1, which may be due to the higher prevalence of cancers with Cyclin D1 than with Cyclin E1 overexpression.

We have previously shown that treatment of mice with PRMT5 inhibitor HLCL65 prevents and suppresses established clinical disease (16). Similarly, T cell-specific PRMT5 deficiency prior to EAE induction prevents the development of EAE (17). Our finding that PRMT5 expression in the cells that are found infiltrating the CNS during EAE correlates with disease severity and the proportion of CD4^+^ T cells in the infiltrating population suggests that T cell PRMT5 contributes to disease severity during the course of the disease. However, the data here provided are correlative, rather than causal. We speculate that PRMT5^+^ T cells in the EAE CNS are proliferating, and the linked Cyclin E1 expression supports this idea. In addition, Cyclin E1 expression was the most highly correlated marker we evaluated to disease severity. Since Cyclin E1 mediates proliferation, it is tempting to speculate that T cell proliferation is an important driver of disease. Cyclin E1 has been linked to EAE in another study, which found that disease suppression by 3,39-Diindolylmethane treatment resulted in low expression of the gene encoding Cyclin E1 (40). It will be interesting to determine whether similar links between Cyclin E1 and disease severity apply to human MS and whether these are modulated from remission to relapse. We plan to address this in future studies.

In summary, our work shows that PRMT5 promotes G1/S cell cycle progression and provide a link between PRMT5 and disease severity and/or progression. Modulating PRMT5 levels may be useful for controlling T cell expansion, and this may help ameliorate diseases linked to T cell activation including MS.

## Data Availability Statement

The raw data supporting the conclusions of this article will be made available by the authors, without undue reservation.

## Ethics Statement

The animal study was reviewed and approved by Institutional Animal Care and Use Committee (IACUC) at The Ohio State University.

## Author Contributions

MG-d-A formulated the hypothesis and designed the experiments. SA and WO performed the experiments and analyzed the data. SA and MG-d-A drafted the manuscript. MG-d-A revised the manuscript. All authors contributed to the article and approved the submitted version.

## Funding

This work was supported by funds from the National Institutes of Health National Institute of Allergy and Infectious Diseases Grants R01AI121405 and 1R21AI127354 (both to MG-d-A), OSUCCC’s Drug Development Institute funds and The Ohio State University School of Health and Rehabilitation Sciences start-up funds (MG-d-A).

## Conflict of Interest

MG-d-A has a PRMT5 inhibitor patent pending and is a PRMT5 inhibitor inventor on a licensing deal with Prelude Therapeutics.

The remaining authors declare that the research was conducted in the absence of any commercial or financial relationships that could be construed as a potential conflict of interest.
