# Supplementary material for: PRMT5 Promotes Cyclin E1 and Cell Cycle Progression in CD4 Th1 Cells and Correlates With EAE Severity
Source: Front Immunol. 2021 Jun 8;12:695947. doi: 10.3389/fimmu.2021.695947 (PMC8217861; doi:10.3389/fimmu.2021.695947)
Supplement: Supplementary file 1 [file DataSheet_1.docx]

Supplemental Figures.


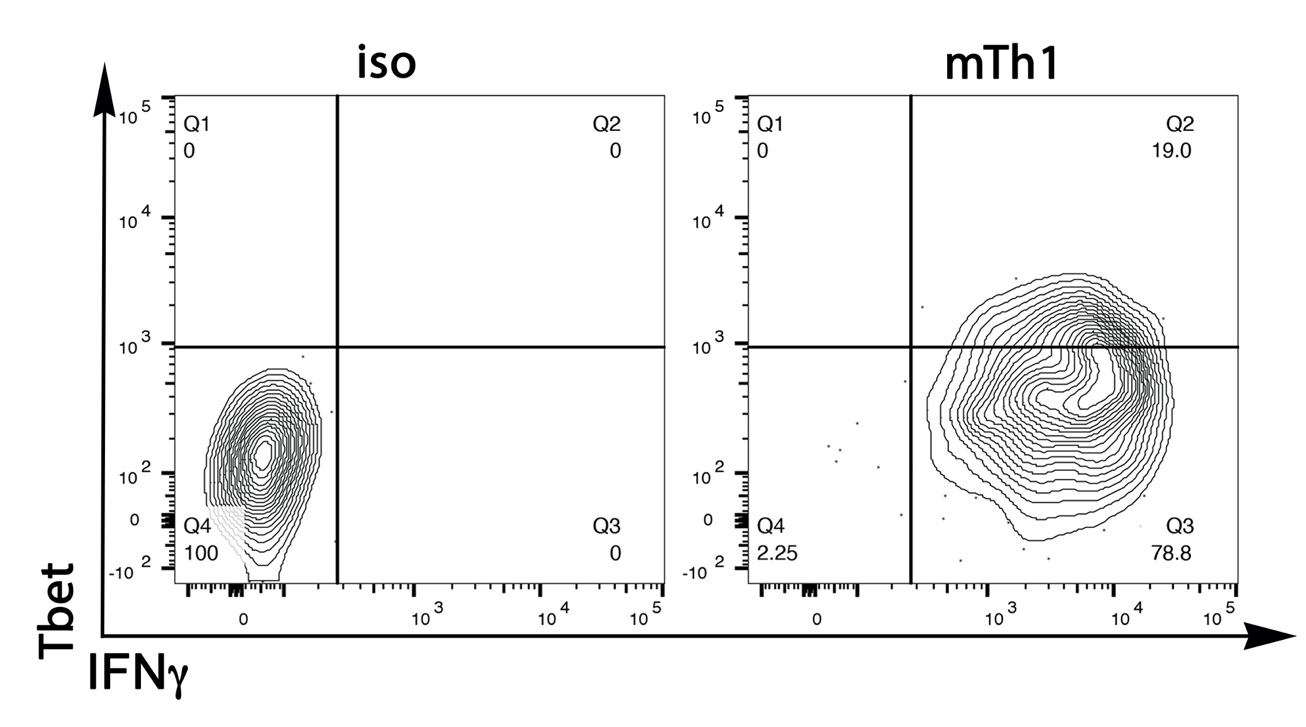


Fig. 1S. Characterization of Th1 differentiated cells. Representative flow cytometry plot of naïve MBP TcR transgenic T cells differentiated in Th1 conditions and then restimulated with MBPAc1-11 and IL-2 for 48 hours. The plot shows live CD4^+^CD44^+^ isotype control (left) and T-bet and Th1 cytokine IFN-γ (right) stained cells, showing approximately 98% of cells were positive.


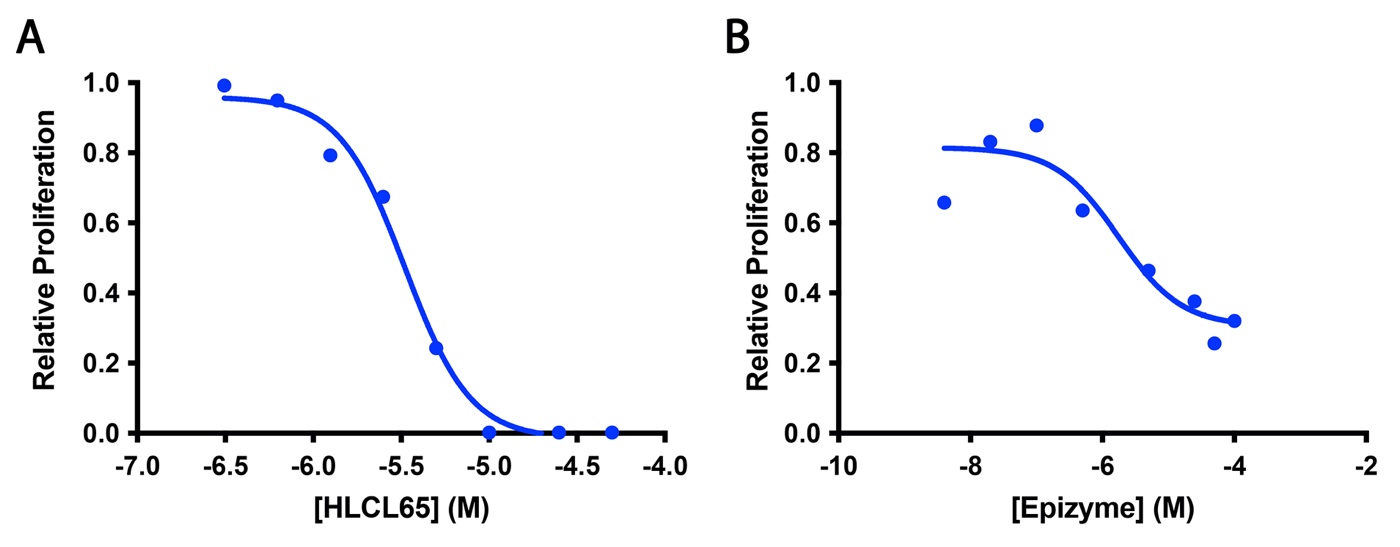


Fig. 2S. Dose response of T cell proliferation inhibition with PRMT5 inhibitors HLCL65 and EPZ015666. MBP TcR transgenic Th1 differentiated cells were activated with anti-CD3/CD28 in the presence of the indicated amounts of PRMT5 inhibitor and their proliferative activity evaluated via tritiated thymidine incorporation.


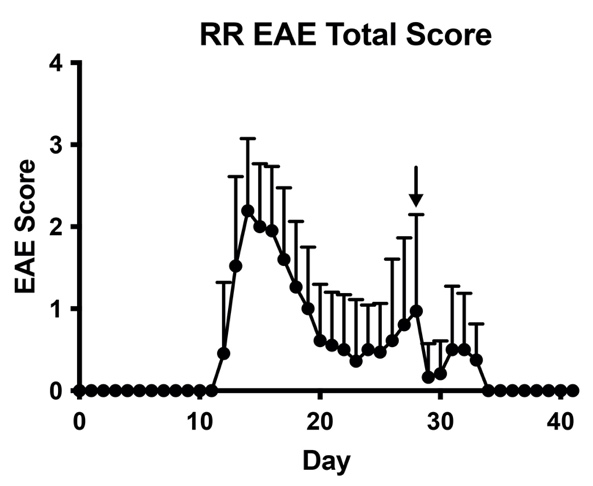


Fig. 3S. EAE scores over time for mice in Fig. 3B-I. SJL mice were immunized as described in Materials and Methods and individual mice were scored for EAE until indicated collection days. Note: due to individual mice being sacrificed for collection and analyses of CNS infiltrating cells, EAE scores appear to artificially dip in the middle of the typical relapse phase (arrow) in this experiment.
